# Supplementary material for: Roles of HIF and 2-Oxoglutarate-Dependent Dioxygenases in Controlling Gene Expression in Hypoxia
Source: Cancers (Basel). 2021 Jan 19;13(2):350. doi: 10.3390/cancers13020350 (PMC7832865; doi:10.3390/cancers13020350)
Supplement: Supplementary file 1 [file cancers-13-00350-s001.pdf]

cancers

MDPI

Supplemental Materials: Roles of HIF and 2-Oxoglutarate-Dependent Dioxygenases in Controlling Gene Expression in Hypoxia

Julianty Frost, Mark Frost, Michael Batie, Hao Jiang and Sonia Rocha

Supplementary Table S1. 2-OGDs reported affinities for oxygen from *in vitro* assays. These Individual studies were used for calculating the median K_M for 2-OGDs with more than one reported value.

Enzyme	O_2 K_M (μM)	Calculated Median K_M
PHD2	67 ± 10 [1]	240
	81 ± 28 [1]	
	229 ± 60 [1]	
	250 [2]	
	Approx. > 450 [3]	
	1746 ± 574 [4]	
FIH	90 ± 20 [5]	110 ± 30
	100 ± 10 [6]	
	0.7-110 [7]	
	110 ± 30 [6]	
	110 ± 73 [1]	
	150 ± 30 [1]	
	237 ± 28 [1]	
KDM4A	57 ± 10 [8]	60 ± 20
	60 ± 20 [9]	
	173 ± 23 [10]	

Supplementary Table S2. Known mutations in the hypoxia signalling system in humans.

Gene	Mutation	Phenotypes
		HIFs
HIF-1α	c.1156C>G (L386V)	Schizophrenia [11]
	c.1744C>T (p.P582S)	Maximal oxygen consumption [12]
	c.1742(p.A588T)	Renal cell carcinoma [13]
HIF-2α	c.607A>C (p.N203H)	Congenital heart disorder [14]
	c.824G>A (p.R275H)	Autism spectrum disorder [15]
	c.1104G>A (p.M368I)	Pheochromocytoma [16]
	c.1121T>A (p.F374Y)	Pheochromocytoma and polycythaemia with paraganglioma [16,17]
	c.1234T>A (p.I412N)	Pheochromocytoma [16]
	c.1235T>A (p.I412N)	Pheochromocytoma [16]
	c.1586T>C (p.L529P)	Paraganglioma, polycythemia and somatostatinoma [18]
	c.1588G>A (p.A530T)	Erythrocytosis and polycythaemia with paraganglioma [19–21]
	c.1589C>A (p.A530E)	Pheochromocytoma [16]
	c.1589C>T (p.A530V)	Polycythaemia with paraganglioma [20]
	c.1591C>T (p.P531S)	Pheochromocytoma and polycythaemia with paraganglioma [20,22]
	c.1591C>A (p.P531T)	Pheochromocytoma and paraganglioma [22]
	c.1592C>T (p.P531L)	Pheochromocytoma and polycythaemia with paraganglioma [16,20,22]
	c.1595A>G (p.Y532C)	Paraganglioma, polycythemia and somatostatinoma [16,18]

	c.1597A>G (p.I533V)	Erythrocytosis [23]
	c.1601C>T (p.P534L)	Erythrocytosis [24]
	c.1604T>C (p.M535T)	Erythrocytosis [25]
	c.1605G>A (p.M535I)	Erythrocytosis [26]
	c.1603A>G (p.M535V)	Erythrocytosis [27]
	c.1609G>A (p.G537R)	Erythrocytosis [28], pulmonary arterial hypertension [29]
	c.1609G>T (p.G537W)	Erythrocytosis [28]
	c.1615G>T (p.D539Y)	Polycythaemia with paraganglioma [20]
	c.1617C>G (p.D539E)	Erythrocytosis [30]
	c.1620C>G (p.F540L)	Erythrocytosis [25]
	c.1625T>C (p.L542P)	Polycythaemia with paraganglioma [31]
	c.2170G>T (p.G724W)	Congenital heart disease [14]
Dioxygenases - hydroxylases		
PHD1	rs10680577	Increased risk of hepatocellular carcinoma [32], lung cancer [33,34], gastric cancer [35], colorectal cancer [36].
	c.188T>A (p.S61R)	Pheochromocytoma/paraganglioma-polycythemia [37]
PHD2	c.12C>A(p.D4E) rs186996510	High-altitude adaptation [38]
	c.380G>C (p.S127C) rs12097901	High-altitude adaptation [38]
	c.471G>C (p.Q157H)	Erythrocytosis [39,40]
	c.599C>A (P200Q)	Erythrocytosis [39]
	c.606delG (p.M2021Ifs*71)	Erythrocytosis [41]
	c.609C>G (p.N203K)	Isolated erythrocytosis [40]
	c.610G>A (p.K204E)	Erythrocytosis [42]
	c.682G>C (p.A228S)	Pheochromocytoma/paraganglioma-polycythemia [37]
	c.760G>C (p.D254H)	Erythrocytosis [39]
	c.799G>A(p.E267K)	Pheochromocytoma [43]
	c.835del14 (p.L279Tfs43*)	Erythrocytosis [44]
	c.840_841insA (p.R281Tfs*3)	Erythrocytosis [41]
	c.853G>C (p.G285R)	Erythrocytosis [42]
	c.872A>T (p.K291I)	Familial isolated erythrocytosis [40]
	c.950C>G (p.P317R)	Familial erythrocytosis [45], cardiopulmonary [46]
	c.1000T>C (p.W334R)	Familial erythrocytosis [47]
	c.1001G>A (p.T334*)	Erythrocytosis [48]
	c.1010dup (p.V338Gfs*18)	Erythrocytosis [42]
	c.1112G>A (p.R371H)	Familial erythrocytosis [39,49]
	c.1121A>G (p.H374R)	Familial erythrocytosis and recurrent paraganglioma [50].
	c.1129C>T (p. Q377*)	Erythrocytosis [41]
	c.1192C>T (p. R398*)	Erythrocytosis [39]
	c.1267A>G (p.K423E)	Isolated erythrocytosis [51]
P4HA1	c.1323_1324insAG (p.R362Gfs*9)	Congenital-onset disorder of connective tissue [52]
	c.1553+2T>G (p.A418_A434del)	
	c.1327A>G (p.K443*)	
P4HA2	c.419A>G (p.Q140R)	High myopia [53]
	c.448A>G (p.I150V)	
	c.871G>A (p.E291K)	
	c.1327A>G (p.K443*)	
	c.1349_1350delGT (p.R451Gfs*8)	
PAHX	c.135-2A>G (p.Y46_R82del)	Refsum disease [54]
	c.164delT (p.L55fs*12)	Refsum disease [55]

	c.244C>G (p.R82G)	Nonsyndromic cleft lip and palate [56]
	c.247A>T (p.N83Y)	Refsum disease [57]
	c.258_265del (p.E86fs*26)	Refsum disease [57]
	c.375_376delGG (p.E126fs*1)	Refsum disease [57]
	c.412_675del (p.E138_W225del)	Refsum disease [58]
	c.457delG (p.A152fs*5)	Refsum disease [57]
	c.497-2A>G (p.A166fs*3)	Refsum disease [57]
	c.517C>T (p.P173S)	Refsum disease [54]
	c.524A>G (p.H175R)	Refsum disease [57]
	c.526C>A (p.Q176K)	Refsum disease [54]
	c.530A>G (p.N177G)	Refsum disease [54]
	c.576_577insGCC (p.192_193insA)	Refsum disease [54]
	c.577T>C (p.W193R)	Refsum disease [54]
	c.589G>C (p.E197Q)	Refsum disease [54]
	c.595A>T (p.I199F)	Refsum disease [54]
	c.610G>A (p.G204S)	Refsum disease [59]
	c.658C>T (p.H220Y)	Refsum disease [54]
	c.678+2T4G (p.A166fs*3)	Refsum disease [57]
	c.678+5G4T (p.A166fs*3)	Refsum disease [57]
	c.679–1G4T (p.A166fs*3)	Refsum disease [57]
	c.683_684insG (p.G228fs*2)	Refsum disease [57]
	c.703G>A (p.G235R)	Refsum disease [60]
	c.734G>A (p.R245Q)	Refsum disease [54], nonsyndromic cleft lip and palate [56]
	c.770T>C (p.F257S)	Refsum disease [54]
	c.805A>C (N269H)	Refsum disease [55]
	c.824G>A (p.R275Q)	Refsum disease [54]
	c.823C>T (p.R275W)	Refsum disease [61]
	c.829C>A (p.A277Q)	Refsum disease [62]
	c.1014_1015insATC (p.N337_L338insH)	Nonsyndromic cleft lip and palate [56]
FIH	c.121C>G (p.P41A)	Colorectal cancer [63]
TET1	NR	
TET2	c.1037T>C (p.L346P)	Myelodysplastic/myeloproliferative disease [64]
	c.1652A>C (p.D551A)	Prostate cancer [65]
	c.4115A>T (p.T1372I)	Myeloproliferative neoplasms [66]
	c.3853_3855del (p.S1285del)	
	p.Q891	
	c.3629T>C(p.L1210P)	
	p.Arg544*	
	splice donor c.3980 + 2G→T	
	p.D1858fs	
	c.3524G>A(p.G1275E)	
TET3	c.1215delA (p.W406Gfs*135)	Intellectual disability, developmental delay, autistic traits, hypotonia, growth abnormalities, facial dysmorphism and movement disorders [67]
	c.2254C>T (p.R752C)	
	c.2552C>T (p.T851M)	
	c.2722G>T (p.V908L)	
	c.3215T>G (p.F1072C)	
	c.3226G>A (p.A1076T)	

	c.3265G>A (p.V1089M)	
	c.4977_4983del (p.H1660Pfs*52)	
	c.5030C>T (p.P1677L)	
	c.5083C>T (p.Q1695*)	
FTO	c.812A>C (p.H271P)	Developmental delay and dysmorphic facial features [68].
	c.947G>A (p.R316Q)	Growth retardation and multiple malformations [68].
	c.956C>T (p.S319F)	Developmental delay and growth retardation [69].
	c.965G>A (p.R322Q)	Growth retardation and multiple malformations [70].
	rs9939609 and rs8050136	Obesity [71].
	rs9939609	Type II diabetes [72]
	rs7202116	Obesity [73].
	rs9939609	Metabolic syndrome including obesity, hypertension, dyslipidemia, and defective glucose tolerance [74].
	rs8050136	
	rs1558902	
	rs1421085	
	Dioxygenases – lysine demethylases	
KDM3A	c.1934C>A (p.P645Q)	Male infertility [75]
	c.3956delA (p.G1211fs)	Male infertility [75]
KDM3B	c.4216C>T (p.R1406W)	Schizophrenia [76]
	c.2624del (p.L875Rfs*8)	Intellectual disability [77]
	c.3422A>G (p.N1141S)	Wilms tumour and hyperpigmentation [78]
	c.916_917delAG	Hepatoblastoma, autism, intellectual disability, and abnormal pigmentation [78]
	c.277G>T (p.E93*)	Acute myeloid leukemia, mild intellectual disability, congenital hypothyroidism and congenital hip dysplasia [78]
	c.3095A>T (p.D1032V)	Hodgkin lymphoma, feeding difficulties, intellectual disability, umbilical and inguinal hernia [79]
	c.133C>T (p.R45*)	Intellectual disability, facial dysmorphism and short stature [79]
	c.277G>T (p.E93*)	
	c.349T>C (p.W117R)	
	c.1007A>G (p.D336G)	
	c.2479C>T (p.Q827*)	
	c.2827C>T (p.R943W)	
	c.2828G>A (p.R943Q)	
	c.3083G>A (p.R1028Q)	
	c.3095A>T (p.D132V)	
	c.4526T>C (p.L1509P)	
	c.4549C>T (p.R1517*)	
	c.4631A>G (p.Y1544C)	
	c.1519G>A (p.E1731K)	
	c.5191G>A (p.E1731K)	
JMJD1C	c.748_749delTT (p.L250fs	Congenital heart disease in patients with 22q11.2 deletion syndrome [80]
	c.488C>T (p.P163L)	Rett syndrome [81]
	c.511G>A (p.V171I)	Intracranial germ cell tumour [82]
	c.268A>G (p.N190D)	Congenital heart disease in patients with 22q11.2 deletion syndrome [80]
	c.860C>G (p.P287R)	Congenital heart disease in patients with 22q11.2 deletion syndrome [80]

	c.1636C>T (p.H546Y)	Congenital heart disease in patients with 22q11.2 deletion syndrome [80]
	c.1957C>T (p.P653S)	Congenital heart disease in patients with 22q11.2 deletion syndrome [80]
	c.2636_2637insTT (p.S880P)	Intracranial germ cell tumour [82]
	c.2822A>G (p.H941R)	Congenital heart disease in patients with 22q11.2 deletion syndrome [80]
	c.2830C>T (p.P944S)	Autism spectrum disorder [81]
	c.3268A>G (p.K1060E)	Intracranial germ cell tumour [82]
	c.3308A>G (p.N1103S)	Intellectual disability [81]
	c.3349A>C (p.I1117L)	Congenital heart disease in patients with 22q11.2 deletion syndrome [80]
	c.3487A>G (p.I1163V)	Autism spectrum disorder [83]
	c.3559A>G (p.T1187A)	Intellectual disability [81]
	c.3743A>G (p.Q1248R)	Intellectual disability [81]
	c.3982C>G (p.R1328G)	Autism spectrum disorder [81]
	c.4286C>T (p.S1429L)	Congenital heart disease in patients with 22q11.2 deletion syndrome [80]
	c.2924G>C (p.K1462N)	Intracranial germ cell tumour [82]
	c.4420T>C (p.S1474P)	Congenital heart disease in patients with 22q11.2 deletion syndrome [80]
	c.4781T>C (p.I1594T)	Intracranial germ cell tumour [82]
	c.6432A>G (p.I2144M)	Intracranial germ cell tumour [82]
	c.6997A>G (p.T2333A)	Autism spectrum disorder [81]
	c.7396C>T (H2466Y)	Congenital heart disease in patients with 22q11.2 deletion syndrome [80]
KDM4C	c.1186G>A (p.D396N)	Upper aerodigestive tract cancer [84]
	c.3115G>A (p.V1039I)	Age at menarche [85]
KDM5A	c.2155C?G (p.R719G)	Intellectual disability [86].
	c.4522C>T (p.R1508W)	Congenital heart disease [87].
KDM5B	c.4109T>G (p.L1370*)	Intellectual disability, dyslexia, global developmental delay, facial dysmorphism [88]
	c.2475-2A>G	Global developmental delay, intellectual disability, aggressive behaviour, facial dysmorphism [88]
	c.895C>T (p.R299*)	
	c.3906delC (p.N1302Kfs*45)	Global developmental delay, hypospadias, facial dysmorphism [88]
	c.622dupT (p.Y208Lfs*5)	
KDM5C	c.2T>C (p.M1T)	X-linked intellectual disability [89]
	c.229G>A (p.A77T)	X-linked mental retardation [90]
	c.260A>G 9p.D87G)	X-linked mental retardation [91]
	c.994C>T (p.R332*)	X-linked mental retardation [91]
	c.1162G>C (p.A388P)	X-linked mental retardation [92]
	c.1204G>T (p.D402Y)	X-linked mental retardation [92]
	c.1270G>T (p.E424*)	Intellectual disability [93]
	c.1353C>G (p.S451R)	X-linked mental retardation [94]
	c.1439C>T (p.P480L)	X-linked intellectual disability [95]
	c.1510G>A (p.V504M)	X-linked mental retardation [90]
	c.1660C>A (p.P554T)	X-linked mental retardation [96]
	c.1919G>A (p.C640Y)	X-linked mental retardation [97]
	c.1924T>C (p.F642L)	X-linked mental retardation [91]

	c.2080C>T (p.R694*)	X-linked mental retardation [92]
	c.2092G>A (p.E698K)	X-linked mental retardation [92]
	c.2152G>C (p.A718P)	Intellectual disability [98]
	c.2172C>A (p.C724*)	X-linked intellectual disability [99]
	c.2191C>T (p.L731F)	X-linked mental retardation [92]
	c.2248C>T (p.R750W)	X-linked mental retardation [91]
	c.2252A>G (p.Y751C)	X-linked mental retardation [91]
	c.2296C>T (p.R766W)	Autism spectrum disorder [100]
	c.2908C>T (p.Q970*)	Intellectual disability [101]
	c.3285C>A (p.C1095*)	Intellectual disability [91]
	c.3864G>A (p.W1288*)	X-linked mental retardation [92]
	IVS11ds+5G>A	X-linked mental retardation [90]
	IVS5ds+2T>C	Intellectual disability [101]
	c.1600delT(p.W534Gfs*15)	Intellectual disability [101]
	c.2047delG (p.A683Pfs*81)	Intellectual disability [91]
	c.3223delG (p.V1075Yfs*2)	X-linked intellectual disability [102]
	c.4441_4442delAG (p.R1481Gfs*9)	X-linked mental retardation [90]
	c.202_203insC (p.68fs*7)	X-linked mental retardation [92]
	c.1296dup (p.E433*)	Intellectual disability [98]
	c.3258_3259insC (p.K1087fs*43)	X-linked mental retardation [96]
	0.4 Mb microdeletion at Xp11.22	Intellectual disability [103]
KDM6A	c.171dupT (p.G58Wfs*7)	Kabuki syndrome [104]
	c.190G>T (p.E64*)	Kabuki syndrome [104]
	c.335−1G>T	Kabuki syndrome [105]
	c.342C>T (p.R172*)	Kabuki syndrome [106,107]
	c.443+5G>C	Kabuki syndrome [104]
	c.563A>G (p.K188R)	Kabuki syndrome [106]
	c.619+6T>C	Kabuki syndrome [104]
	c.620−2A>G	Kabuki syndrome [104]
	c.752G>A (p.W251*)	Kabuki syndrome [108]
	c.1555C>T (p.R519*)	Kabuki syndrome [109]
	c.1834C>T (p.R612*)	Renal cancer [110]
	c.1846_1849delACTC (p.T616Yfs*8)	Kabuki syndrome [107]
	c.1909_1912delTCTA (p.S637Tfs*53)	Kabuki syndrome [111]
	c.2226_2227dupCA (p.S743Tfs*13)	Kabuki syndrome [104]
	c.2515_2518del (p.N839Vfs*27)	Kabuki syndrome [112]
	c.2729A>G (p.N910S)	Kabuki syndrome [104]
	c.2832+1G>A	Kabuki syndrome [104]
	c.2839A>T (p.D980V)	Kabuki syndrome [107]
	c.3073A>G (p.S1025G)	Kabuki syndrome [104]
	c.3109C>T (p.Q1037*)	Kabuki syndrome [104]
	c.3284+3_3284+6delAAGT (p.N1070_K1094del)	Kabuki syndrome [107]
	c.3354_3356delTCT (p.L1119del)	Kabuki syndrome [111]
	c.3717G>A (p.W1239*)	Kabuki syndrome [111]

	c.3763C>T (p.R1255W)	Kabuki syndrome [104]
	c.3835C>T (p.R1279*)	Kabuki syndrome [113]
	c.4051C>T (p.R1351*)	Kabuki syndrome [109]
	c.3284+1G>T	Kabuki syndrome [106]
	c.3548+2T>C	Kabuki syndrome [106]
	Exon 6 deletion	Kabuki syndrome [106]
	site<br>c.3878+3_3878+6delAAGT<br>c.3878+3_3878+6delAAGT	Kabuki syndrome [106]
	c.3501delT (p.F1167Lfs*11)	Kabuki syndrome [106]
	c.3736+2T>C	Kabuki syndrome [108]
	c. 3835C>T (p.R1279*)	Biliary atresia with Kabuki syndrome-like features [114]
	c.3876_3878delTAA+1delG	Kabuki syndrome [115]
KDM6B	c.2661C>T (p.P888S)	Intellectual disability [86]
	IVS9ds+5G>T	Intellectual disability, brachydactyly and dysmorphism [116]
KDM7B	c.529A>T (p.K177*)	X-linked mental retardation with cleft lip/palate [117]
	c.631C>T (p.R211*)	X-linked mental retardation with cleft lip/palate [118]
	c.836T>C (p.F279S)	X-linked mental retardation with cleft lip/palate [119]
	p.S969del	Autism and Asperger syndrome [120]
	c.943_954del	X-linked mental retardation with cleft lip/palate [118]
	g.218-254 kb covering KDM7B	Autism spectrum disorder, intellectual disability, cleft palate and Aarskog syndrome [121]
	g.236,505 bp	Intellectual disability [101]

NR, not reported.

References

1. Ehrismann, D.; Flashman, E.; Genn, D.N.; Mathioudakis, N.; Hewitson, K.S.; Ratcliffe, P.J.; Schofield, C.J. Studies on the activity of the hypoxia-inducible-factor hydroxylases using an oxygen consumption assay. *Biochem. J.* **2007**, *401*, 227–234, doi:10.1042/BJ20061151.
2. Hirsila, M.; Koivunen, P.; Gunzler, V.; Kivirikko, K.I.; Myllyharju, J. Characterization of the human prolyl 4-hydroxylases that modify the hypoxia-inducible factor. *J. Biol. Chem.* **2003**, *278*, 30772–30780, doi:10.1074/jbc.M304982200.
3. Tarhonskaya, H.; Chowdhury, R.; Leung, I.K.; Loik, N.D.; McCullagh, J.S.; Claridge, T.D.; Schofield, C.J.; Flashman, E. Investigating the contribution of the active site environment to the slow reaction of hypoxia-inducible factor prolyl hydroxylase domain 2 with oxygen. *Biochem. J.* **2014**, *463*, 363–372, doi:10.1042/BJ20140779.
4. Dao, J.H.; Kurzeja, R.J.; Morachis, J.M.; Veith, H.; Lewis, J.; Yu, V.; Tegley, C.M.; Tagari, P. Kinetic characterization and identification of a novel inhibitor of hypoxia-inducible factor prolyl hydroxylase 2 using a time-resolved fluorescence resonance energy transfer-based assay technology. *Anal. Biochem.* **2009**, *384*, 213–223, doi:10.1016/j.ab.2008.09.052.
5. Koivunen, P.; Hirsila, M.; Gunzler, V.; Kivirikko, K.I.; Myllyharju, J. Catalytic properties of the asparaginyl hydroxylase (FIH) in the oxygen sensing pathway are distinct from those of its prolyl 4-hydroxylases. *J. Biol. Chem.* **2004**, *279*, 9899–9904, doi:10.1074/jbc.M312254200.
6. Tarhonskaya, H.; Hardy, A.P.; Howe, E.A.; Loik, N.D.; Kramer, H.B.; McCullagh, J.S.; Schofield, C.J.; Flashman, E. Kinetic Investigations of the Role of Factor Inhibiting Hypoxia-inducible Factor (FIH) as an Oxygen Sensor. *J. Biol. Chem.* **2015**, *290*, 19726–19742, doi:10.1074/jbc.M115.653014.
7. Wilkins, S.E.; Hyvarinen, J.; Chicher, J.; Gorman, J.J.; Peet, D.J.; Bilton, R.L.; Koivunen, P. Differences in hydroxylation and binding of Notch and HIF-1alpha demonstrate substrate selectivity for factor inhibiting HIF-1 (FIH-1). *Int. J. Biochem Cell Biol.* **2009**, *41*, 1563–1571, doi:10.1016/j.biocel.2009.01.005.
8. Hancock, R.L.; Masson, N.; Dunne, K.; Flashman, E.; Kawamura, A. The Activity of JmjC Histone Lysine Demethylase KDM4A is Highly Sensitive to Oxygen Concentrations. *ACS Chem. Biol.* **2017**, *12*, 1011–1019, doi:10.1021/acschembio.6b00958.
9. Chakraborty, A.A.; Laukka, T.; Myllykoski, M.; Ringel, A.E.; Booker, M.A.; Tolstorukov, M.Y.; Meng, Y.J.; Meier, S.R.; Jennings, R.B.; Creech, A.L., et al. Histone demethylase KDM6A directly senses oxygen to control chromatin and cell fate. *Science* **2019**, *363*, 1217–1222, doi:10.1126/science.aaw1026.
10. Cascella, B.; Mirica, L.M. Kinetic analysis of iron-dependent histone demethylases: alpha-ketoglutarate substrate inhibition and potential relevance to the regulation of histone demethylation in cancer cells. *Biochemistry* **2012**, *51*, 8699–8701, doi:10.1021/bi3012466.

11. Gulsuner, S.; Walsh, T.; Watts, A.C.; Lee, M.K.; Thornton, A.M.; Casadei, S.; Rippey, C.; Shahin, H.; Consortium on the Genetics of, S.; Group, P.S., et al. Spatial and temporal mapping of de novo mutations in schizophrenia to a fetal prefrontal cortical network. *Cell* **2013**, *154*, 518–529, doi:10.1016/j.cell.2013.06.049.
12. Prior, S.J.; Hagberg, J.M.; Phares, D.A.; Brown, M.D.; Fairfull, L.; Ferrell, R.E.; Roth, S.M. Sequence variation in hypoxia-inducible factor 1alpha (HIF1A): association with maximal oxygen consumption. *Physiol. Genomics* **2003**, *15*, 20–26, doi:10.1152/physiolgenomics.00061.2003.
13. Ollerenshaw, M.; Page, T.; Hammonds, J.; Demaine, A. Polymorphisms in the hypoxia inducible factor-1alpha gene (HIF1A) are associated with the renal cell carcinoma phenotype. *Cancer Genet. Cytogenet.* **2004**, *153*, 122–126, doi:10.1016/j.cancergencyto.2004.01.014.
14. Pan, H.; Chen, Q.; Qi, S.; Li, T.; Liu, B.; Liu, S.; Ma, X.; Wang, B. Mutations in EPAS1 in congenital heart disease in Tibetans. *Biosci. Rep.* **2018**, *38*, doi:10.1042/BSR20181389.
15. An, J.Y.; Cristino, A.S.; Zhao, Q.; Edson, J.; Williams, S.M.; Ravine, D.; Wray, J.; Marshall, V.M.; Hunt, A.; Whitehouse, A.J., et al. Towards a molecular characterization of autism spectrum disorders: an exome sequencing and systems approach. *Transl. Psychiatry* **2014**, *4*, e394, doi:10.1038/tp.2014.38.
16. Welander, J.; Andreasson, A.; Brauckhoff, M.; Backdahl, M.; Larsson, C.; Gimm, O.; Soderkvist, P. Frequent EPAS1/HIF2alpha exons 9 and 12 mutations in non-familial pheochromocytoma. *Endocr. Relat. Cancer* **2014**, *21*, 495–504, doi:10.1530/ERC-13-0384.
17. Lorenzo, F.R.; Yang, C.; Ng Tang Fui, M.; Vankayalapati, H.; Zhuang, Z.; Huynh, T.; Grossmann, M.; Pacak, K.; Prchal, J.T. A novel EPAS1/HIF2A germline mutation in a congenital polycythemia with paraganglioma. *J. Mol. Med. (Berl)* **2013**, *91*, 507–512, doi:10.1007/s00109-012-0967-z.
18. Yang, C.; Sun, M.G.; Matro, J.; Huynh, T.T.; Rahimpour, S.; Prchal, J.T.; Lechan, R.; Lonser, R.; Pacak, K.; Zhuang, Z. Novel HIF2A mutations disrupt oxygen sensing, leading to polycythemia, paragangliomas, and somatostatinomas. *Blood* **2013**, *121*, 2563–2566, doi:10.1182/blood-2012-10-460972.
19. Yang, C.; Hong, C.S.; Prchal, J.T.; Balint, M.T.; Pacak, K.; Zhuang, Z. Somatic mosaicism of EPAS1 mutations in the syndrome of paraganglioma and somatostatinoma associated with polycythemia. *Hum. Genome. Var.* **2015**, *2*, 15053, doi:10.1038/hgv.2015.53.
20. Comino-Mendez, I.; de Cubas, A.A.; Bernal, C.; Alvarez-Escola, C.; Sanchez-Malo, C.; Ramirez-Tortosa, C.L.; Pedrinaci, S.; Rapizzi, E.; Ercolino, T.; Bernini, G., et al. Tumoral EPAS1 (HIF2A) mutations explain sporadic pheochromocytoma and paraganglioma in the absence of erythrocytosis. *Hum. Mol. Genet.* **2013**, *22*, 2169–2176, doi:10.1093/hmg/ddt069.
21. Zhuang, Z.; Yang, C.; Lorenzo, F.; Merino, M.; Fojo, T.; Kebebew, E.; Popovic, V.; Stratakis, C.A.; Prchal, J.T.; Pacak, K. Somatic HIF2A gain-of-function mutations in paraganglioma with polycythemia. *N. Engl. J. Med.* **2012**, *367*, 922–930, doi:10.1056/NEJMoa1205119.
22. Toledo, R.A.; Qin, Y.; Srikantan, S.; Morales, N.P.; Li, Q.; Deng, Y.; Kim, S.W.; Pereira, M.A.; Toledo, S.P.; Su, X., et al. In vivo and in vitro oncogenic effects of HIF2A mutations in pheochromocytomas and paragangliomas. *Endocr. Relat. Cancer* **2013**, *20*, 349–359, doi:10.1530/ERC-13-0101.
23. Perrotta, S.; Stiehl, D.P.; Punzo, F.; Scianguetta, S.; Borriello, A.; Bencivenga, D.; Casale, M.; Nobili, B.; Fasoli, S.; Balduzzi, A., et al. Congenital erythrocytosis associated with gain-of-function HIF2A gene mutations and erythropoietin levels in the normal range. *Haematologica* **2013**, *98*, 1624–1632, doi:10.3324/haematol.2013.088369.
24. Furlow, P.W.; Percy, M.J.; Sutherland, S.; Bierl, C.; McMullin, M.F.; Master, S.R.; Lappin, T.R.; Lee, F.S. Erythrocytosis-associated HIF-2alpha mutations demonstrate a critical role for residues C-terminal to the hydroxylacceptor proline. *J. Biol. Chem.* **2009**, *284*, 9050–9058, doi:10.1074/jbc.M808737200.
25. Percy, M.J.; Chung, Y.J.; Harrison, C.; Mercieca, J.; Hoffbrand, A.V.; Dinardo, C.L.; Santos, P.C.; Fonseca, G.H.; Gualandro, S.F.; Pereira, A.C., et al. Two new mutations in the HIF2A gene associated with erythrocytosis. *Am. J. Hematol.* **2012**, *87*, 439–442, doi:10.1002/ajh.23123.
26. Martini, M.; Teofili, L.; Cenci, T.; Giona, F.; Torti, L.; Rea, M.; Foa, R.; Leone, G.; Larocca, L.M. A novel heterozygous HIF2AM535I mutation reinforces the role of oxygen sensing pathway disturbances in the pathogenesis of familial erythrocytosis. *Haematologica* **2008**, *93*, 1068–1071, doi:10.3324/haematol.13210.
27. Percy, M.J.; Beer, P.A.; Campbell, G.; Dekker, A.W.; Green, A.R.; Oscier, D.; Rainey, M.G.; van Wijk, R.; Wood, M.; Lappin, T.R., et al. Novel exon 12 mutations in the HIF2A gene associated with erythrocytosis. *Blood* **2008**, *111*, 5400–5402, doi:10.1182/blood-2008-02-137703.
28. Percy, M.J.; Furlow, P.W.; Lucas, G.S.; Li, X.; Lappin, T.R.; McMullin, M.F.; Lee, F.S. A gain-of-function mutation in the HIF2A gene in familial erythrocytosis. *N. Engl. J. Med.* **2008**, *358*, 162–168, doi:10.1056/NEJMoa073123.
29. Gale, D.P.; Harten, S.K.; Reid, C.D.; Tuddenham, E.G.; Maxwell, P.H. Autosomal dominant erythrocytosis and pulmonary arterial hypertension associated with an activating HIF2 alpha mutation. *Blood* **2008**, *112*, 919–921, doi:10.1182/blood-2008-04-153718.
30. van Wijk, R.; Sutherland, S.; Van Wesel, A.C.; Huizinga, E.G.; Percy, M.J.; Bierings, M.; Lee, F.S. Erythrocytosis associated with a novel missense mutation in the HIF2A gene. *Haematologica* **2010**, *95*, 829–832, doi:10.3324/haematol.2009.017582.
31. Buffet, A.; Smati, S.; Mansuy, L.; Menara, M.; Lebras, M.; Heymann, M.F.; Simian, C.; Favier, J.; Murat, A.; Cariou, B., et al. Mosaicism in HIF2A-related polycythemia-paraganglioma syndrome. *J. Clin. Endocrinol. Metab.* **2014**, *99*, E369–373, doi:10.1210/jc.2013-2600.

32. Zhu, Z.; Gao, X.; He, Y.; Zhao, H.; Yu, Q.; Jiang, D.; Zhang, P.; Ma, X.; Huang, H.; Dong, D., et al. An insertion/deletion polymorphism within RERT-lncRNA modulates hepatocellular carcinoma risk. *Cancer Res.* **2012**, *72*, 6163–6172, doi:10.1158/0008-5472.CAN-12-0010.
33. Che, J.; Jiang, D.; Zheng, Y.; Zhu, B.; Zhang, P.; Lu, D.; Zhang, J.; Xiao, J.; Wang, J.; Gao, Y., et al. Polymorphism in PHD1 gene and risk of non-small cell lung cancer in a Chinese population. *Tumour. Biol.* **2014**, *35*, 8921–8925, doi:10.1007/s13277-014-2112-9.
34. Zhu, J.; Luo, J.Z.; Li, C.B. Correlations of an Insertion/Deletion Polymorphism (rs10680577) in the RERT-lncRNA with the Susceptibility, Clinicopathological Features, and Prognosis of Lung Cancer. *Biochem. Genet.* **2019**, *57*, 147–158, doi:10.1007/s10528-018-9883-4.
35. Wang, J.; Zhang, J.; Zhou, C.; Chen, L.; Yu, Q. An insertion/deletion polymorphism within the proximal promoter of EGLN2 is associated with susceptibility for gastric cancer in the Chinese population. *Genet. Test. Mol. Biomarkers* **2014**, *18*, 269–273, doi:10.1089/gtmb.2013.0438.
36. Li, C.; Feng, L.; Niu, L.; Teng Li, T.; Zhang, B.; Wan, H.; Zhu, Z.; Liu, H.; Wang, K.; Fu, H., et al. An insertion/deletion polymorphism within the promoter of EGLN2 is associated with susceptibility to colorectal cancer. *Int. J. Biol. Markers* **2017**, *32*, e274–e277, doi:10.5301/jbm.5000253.
37. Yang, C.; Zhuang, Z.; Fliedner, S.M.; Shankavaram, U.; Sun, M.G.; Bullova, P.; Zhu, R.; Elkahloun, A.G.; Kourlas, P.J.; Merino, M., et al. Germ-line PHD1 and PHD2 mutations detected in patients with pheochromocytoma/paraganglioma-polycythemia. *J. Mol. Med. (Berl)* **2015**, *93*, 93–104, doi:10.1007/s00109-014-1205-7.
38. Xiang, K.; Ouzhuluobu; Peng, Y.; Yang, Z.; Zhang, X.; Cui, C.; Zhang, H.; Li, M.; Zhang, Y.; Bianba, et al. Identification of a Tibetan-specific mutation in the hypoxic gene EGLN1 and its contribution to high-altitude adaptation. *Mol. Biol. Evol.* **2013**, *30*, 1889–1898, doi:10.1093/molbev/mst090.
39. Ladroue, C.; Hoogewijs, D.; Gad, S.; Carcenac, R.; Storti, F.; Barrois, M.; Gimenez-Roqueplo, A.P.; Leporrier, M.; Casadevall, N.; Hermine, O., et al. Distinct deregulation of the hypoxia inducible factor by PHD2 mutants identified in germline DNA of patients with polycythemia. *Haematologica* **2012**, *97*, 9–14, doi:10.3324/haematol.2011.044644.
40. Albiero, E.; Ruggeri, M.; Fortuna, S.; Bernardi, M.; Finotto, S.; Madeo, D.; Rodeghiero, F. Analysis of the oxygen sensing pathway genes in familial chronic myeloproliferative neoplasms and identification of a novel EGLN1 germ-line mutation. *Br. J. Haematol.* **2011**, *153*, 405–408, doi:10.1111/j.1365-2141.2010.08551.x.
41. Al-Sheikh, M.; Moradkhani, K.; Lopez, M.; Wajcman, H.; Prehu, C. Disturbance in the HIF-1alpha pathway associated with erythrocytosis: further evidences brought by frameshift and nonsense mutations in the prolyl hydroxylase domain protein 2 (PHD2) gene. *Blood Cells Mol. Dis.* **2008**, *40*, 160–165, doi:10.1016/j.bcmd.2007.07.017.
42. Bento, C.; Percy, M.J.; Gardie, B.; Maia, T.M.; van Wijk, R.; Perrotta, S.; Della Ragione, F.; Almeida, H.; Rossi, C.; Girodon, F., et al. Genetic basis of congenital erythrocytosis: mutation update and online databases. *Hum. Mutat.* **2014**, *35*, 15–26, doi:10.1002/humu.22448.
43. Welander, J.; Andreasson, A.; Juhlin, C.C.; Wiseman, R.W.; Backdahl, M.; Hoog, A.; Larsson, C.; Gimm, O.; Soderkvist, P. Rare germline mutations identified by targeted next-generation sequencing of susceptibility genes in pheochromocytoma and paraganglioma. *J. Clin. Endocrinol. Metab.* **2014**, *99*, E1352–1360, doi:10.1210/jc.2013-4375.
44. Jang, J.H.; Seo, J.Y.; Jang, J.; Jung, C.W.; Lee, K.O.; Kim, S.H.; Kim, H.J. Hereditary gene mutations in Korean patients with isolated erythrocytosis. *Ann. Hematol.* **2014**, *93*, 931–935, doi:10.1007/s00277-014-2006-3.
45. Percy, M.J.; Zhao, Q.; Flores, A.; Harrison, C.; Lappin, T.R.; Maxwell, P.H.; McMullin, M.F.; Lee, F.S. A family with erythrocytosis establishes a role for prolyl hydroxylase domain protein 2 in oxygen homeostasis. *Proc. Natl. Acad. Sci. U S A* **2006**, *103*, 654–659, doi:10.1073/pnas.0508423103.
46. Talbot, N.P.; Smith, T.G.; Balanos, G.M.; Dorrington, K.L.; Maxwell, P.H.; Robbins, P.A. Cardiopulmonary phenotype associated with human PHD2 mutation. *Physiol. Rep.* **2017**, *5*, doi:10.14814/phy2.13224.
47. Bento, C.; Almeida, H.; Maia, T.M.; Relvas, L.; Oliveira, A.C.; Rossi, C.; Girodon, F.; Fernandez-Lago, C.; Aguado-Diaz, A.; Fraga, C., et al. Molecular study of congenital erythrocytosis in 70 unrelated patients revealed a potential causal mutation in less than half of the cases (Where is/are the missing gene(s)?). *Eur. J. Haematol.* **2013**, *91*, 361–368, doi:10.1111/ejh.12170.
48. Wilson, R.; Syed, N.; Shah, P. Erythrocytosis due to PHD2 Mutations: A Review of Clinical Presentation, Diagnosis, and Genetics. *Case Rep. Hematol* **2016**, doi:10.1155/2016/6373706.
49. Percy, M.J.; Furlow, P.W.; Beer, P.A.; Lappin, T.R.; McMullin, M.F.; Lee, F.S. A novel erythrocytosis-associated PHD2 mutation suggests the location of a HIF binding groove. *Blood* **2007**, *110*, 2193–2196, doi:10.1182/blood-2007-04-084434.
50. Ladroue, C.; Carcenac, R.; Leporrier, M.; Gad, S.; Le Hello, C.; Galateau-Salle, F.; Feunteun, J.; Pouyssegur, J.; Richard, S.; Gardie, B. PHD2 mutation and congenital erythrocytosis with paraganglioma. *N. Engl. J. Med.* **2008**, *359*, 2685–2692, doi:10.1056/NEJMoa0806277.
51. Albiero, E.; Ruggeri, M.; Fortuna, S.; Finotto, S.; Bernardi, M.; Madeo, D.; Rodeghiero, F. Isolated erythrocytosis: study of 67 patients and identification of three novel germ-line mutations in the prolyl hydroxylase domain protein 2 (PHD2) gene. *Haematologica* **2012**, *97*, 123–127, doi:10.3324/haematol.2010.039545.
52. Zou, Y.; Donkervoort, S.; Salo, A.M.; Foley, A.R.; Barnes, A.M.; Hu, Y.; Makareeva, E.; Leach, M.E.; Mohassel, P.; Dastgir, J., et al. P4HA1 mutations cause a unique congenital disorder of connective tissue involving tendon, bone, muscle and the eye. *Hum. Mol. Genet.* **2017**, *26*, 2207–2217, doi:10.1093/hmg/ddx110.

53. Guo, H.; Tong, P.; Liu, Y.; Xia, L.; Wang, T.; Tian, Q.; Li, Y.; Hu, Y.; Zheng, Y.; Jin, X., et al. Mutations of P4HA2 encoding prolyl 4-hydroxylase 2 are associated with nonsyndromic high myopia. *Genet. Med.* **2015**, *17*, 300–306, doi:10.1038/gim.2015.28.
54. Jansen, G.A.; Hogenhout, E.M.; Ferdinandusse, S.; Waterham, H.R.; Ofman, R.; Jakobs, C.; Skjeldal, O.H.; Wanders, R.J. Human phytanoyl-CoA hydroxylase: resolution of the gene structure and the molecular basis of Refsum's disease. *Hum. Mol. Genet.* **2000**, *9*, 1195–1200, doi:10.1093/hmg/9.8.1195.
55. Jansen, G.A.; Ofman, R.; Ferdinandusse, S.; Ijlst, L.; Muijsers, A.O.; Skjeldal, O.H.; Stokke, O.; Jakobs, C.; Besley, G.T.; Wraith, J.E., et al. Refsum disease is caused by mutations in the phytanoyl-CoA hydroxylase gene. *Nat. Genet.* **1997**, *17*, 190–193, doi:10.1038/ng1097-190.
56. Aylward, A.; Cai, Y.; Lee, A.; Blue, E.; Rabinowitz, D.; Haddad, J., Jr.; University of Washington Center for Mendelian, G. Using Whole Exome Sequencing to Identify Candidate Genes With Rare Variants In Nonsyndromic Cleft Lip and Palate. *Genet. Epidemiol.* **2016**, *40*, 432–441, doi:10.1002/gepi.21972.
57. Jansen, G.A.; Waterham, H.R.; Wanders, R.J. Molecular basis of Refsum disease: sequence variations in phytanoyl-CoA hydroxylase (PHYH) and the PTS2 receptor (PEX7). *Hum. Mutat.* **2004**, *23*, 209–218, doi:10.1002/humu.10315.
58. Chahal, A.; Khan, M.; Pai, S.G.; Barbosa, E.; Singh, I. Restoration of phytanic acid oxidation in Refsum disease fibroblasts from patients with mutations in the phytanoyl-CoA hydroxylase gene. *FEBS Lett.* **1998**, *429*, 119–122, doi:10.1016/s0014-5793(98)00575-4.
59. Jansen, G.A.; Ferdinandusse, S.; Skjeldal, O.H.; Stokke, O.; de Groot, C.J.; Jakobs, C.; Wanders, R.J. Molecular basis of Refsum disease: identification of new mutations in the phytanoyl-CoA hydroxylase cDNA. *J. Inherit. Metab. Dis.* **1998**, *21*, 288–291, doi:10.1023/a:1005388710197.
60. Zhao, L.; Wang, F.; Wang, H.; Li, Y.; Alexander, S.; Wang, K.; Willoughby, C.E.; Zaneveld, J.E.; Jiang, L.; Soens, Z.T., et al. Next-generation sequencing-based molecular diagnosis of 82 retinitis pigmentosa probands from Northern Ireland. *Hum. Genet.* **2015**, *134*, 217–230, doi:10.1007/s00439-014-1512-7.
61. Mihalik, S.J.; Morrell, J.C.; Kim, D.; Sacksteder, K.A.; Watkins, P.A.; Gould, S.J. Identification of PAHX, a Refsum disease gene. *Nat. Genet.* **1997**, *17*, 185–189, doi:10.1038/ng1097-185.
62. Kohlschutter, A.; Santer, R.; Lukacs, Z.; Altenburg, C.; Kemper, M.J.; Ruther, K. A child with night blindness: preventing serious symptoms of Refsum disease. *J. Child. Neurol.* **2012**, *27*, 654–656, doi:10.1177/0883073811424799.
63. Webb, E.L.; Rudd, M.F.; Sellick, G.S.; El Galta, R.; Bethke, L.; Wood, W.; Fletcher, O.; Penegar, S.; Withey, L.; Qureshi, M., et al. Search for low penetrance alleles for colorectal cancer through a scan of 1467 non-synonymous SNPs in 2575 cases and 2707 controls with validation by kin-cohort analysis of 14 704 first-degree relatives. *Hum. Mol. Genet.* **2006**, *15*, 3263–3271, doi:10.1093/hmg/ddl401.
64. Ismael, O.; Shimada, A.; Hama, A.; Elshazley, M.; Muramatsu, H.; Goto, A.; Sakaguchi, H.; Tanaka, M.; Takahashi, Y.; Yinyan, X., et al. De novo childhood myelodysplastic/myeloproliferative disease with unique molecular characteristics. *Br. J. Haematol* **2012**, *158*, 129–137, doi:10.1111/j.1365-2141.2012.09140.x.
65. Nickerson, M.L.; Im, K.M.; Misner, K.J.; Tan, W.; Lou, H.; Gold, B.; Wells, D.W.; Bravo, H.C.; Fredrikson, K.M.; Harkins, T.T., et al. Somatic alterations contributing to metastasis of a castration-resistant prostate cancer. *Hum. Mutat.* **2013**, *34*, 1231–1241, doi:10.1002/humu.22346.
66. Schaub, F.X.; Looser, R.; Li, S.; Hao-Shen, H.; Lehmann, T.; Tichelli, A.; Skoda, R.C. Clonal analysis of TET2 and JAK2 mutations suggests that TET2 can be a late event in the progression of myeloproliferative neoplasms. *Blood* **2010**, *115*, 2003–2007, doi:10.1182/blood-2009-09-245381.
67. Beck, D.B.; Petracovici, A.; He, C.; Moore, H.W.; Louie, R.J.; Ansar, M.; Douzgou, S.; Sithambaram, S.; Cottrell, T.; Santos-Cortez, R.L.P., et al. Delineation of a Human Mendelian Disorder of the DNA Demethylation Machinery: TET3 Deficiency. *Am. J. Hum. Genet.* **2020**, *106*, 234–245, doi:10.1016/j.ajhg.2019.12.007.
68. Caglayan, A.O.; Tuysuz, B.; Coskun, S.; Quon, J.; Harmanci, A.S.; Baranoski, J.F.; Baran, B.; Erson-Omay, E.Z.; Henegariu, O.; Mane, S.M., et al. A patient with a novel homozygous missense mutation in FTO and concomitant nonsense mutation in CETP. *J. Hum. Genet.* **2016**, *61*, 395–403, doi:10.1038/jhg.2015.160.
69. Daoud, H.; Zhang, D.; McMurray, F.; Yu, A.; Luco, S.M.; Vanstone, J.; Jarinova, O.; Carson, N.; Wickens, J.; Shishodia, S., et al. Identification of a pathogenic FTO mutation by next-generation sequencing in a newborn with growth retardation and developmental delay. *J. Med. Genet.* **2016**, *53*, 200–207, doi:10.1136/jmedgenet-2015-103399.
70. Rohena, L.; Lawson, M.; Guzman, E.; Ganapathi, M.; Cho, M.T.; Haverfield, E.; Anyane-Yeboa, K. FTO variant associated with malformation syndrome. *Am. J. Med. Genet. A* **2016**, *170A*, 1023–1028, doi:10.1002/ajmg.a.37515.
71. Song, Y.; You, N.C.; Hsu, Y.H.; Howard, B.V.; Langer, R.D.; Manson, J.E.; Nathan, L.; Niu, T.; L, F.T.; Liu, S. FTO polymorphisms are associated with obesity but not diabetes risk in postmenopausal women. *Obesity (Silver Spring)* **2008**, *16*, 2472–2480, doi:10.1038/oby.2008.408.
72. Frayling, T.M.; Timpson, N.J.; Weedon, M.N.; Zeggini, E.; Freathy, R.M.; Lindgren, C.M.; Perry, J.R.; Elliott, K.S.; Lango, H.; Rayner, N.W., et al. A common variant in the FTO gene is associated with body mass index and predisposes to childhood and adult obesity. *Science* **2007**, *316*, 889–894, doi:10.1126/science.1141634.
73. Yang, J.; Loos, R.J.; Powell, J.E.; Medland, S.E.; Speliotes, E.K.; Chasman, D.I.; Rose, L.M.; Thorleifsson, G.; Steinthorsdottir, V.; Magi, R., et al. FTO genotype is associated with phenotypic variability of body mass index. *Nature* **2012**, *490*, 267–272, doi:10.1038/nature11401.

74. Hotta, K.; Kitamoto, T.; Kitamoto, A.; Mizusawa, S.; Matsuo, T.; Nakata, Y.; Kamohara, S.; Miyatake, N.; Kotani, K.; Komatsu, R., et al. Association of variations in the FTO, SCG3 and MTMR9 genes with metabolic syndrome in a Japanese population. *J. Hum. Genet.* **2011**, *56*, 647–651, doi:10.1038/jhg.2011.74.
75. Hojati, Z.; Soleimanpour, E.; Javadirad, S.M.; Nasr-Esfahani, M.H. Identification of Two Novel Mutations in KDM3A Regulatory Gene in Iranian Infertile Males. *Iran. Biomed. J.* **2019**, *23*, 220–227.
76. Guipponi, M.; Santoni, F.A.; Setola, V.; Gehrig, C.; Rotharmel, M.; Cuenca, M.; Guillin, O.; Dikeos, D.; Georgantopoulos, G.; Papadimitriou, G., et al. Exome sequencing in 53 sporadic cases of schizophrenia identifies 18 putative candidate genes. *PLoS One* **2014**, *9*, e112745, doi:10.1371/journal.pone.0112745.
77. Deciphering Developmental Disorders, S. Prevalence and architecture of de novo mutations in developmental disorders. *Nature* **2017**, *542*, 433–438, doi:10.1038/nature21062.
78. Mahamdallie, S.; Yost, S.; Poyastro-Pearson, E.; Holt, E.; Zachariou, A.; Seal, S.; Elliott, A.; Clarke, M.; Warren-Perry, M.; Hanks, S., et al. Identification of new Wilms tumour predisposition genes: an exome sequencing study. *Lancet Child. Adolesc. Health* **2019**, *3*, 322–331, doi:10.1016/S2352-4642(19)30018-5.
79. Diets, I.J.; van der Donk, R.; Baltrunaite, K.; Waanders, E.; Reijnders, M.R.F.; Dingemans, A.J.M.; Pfundt, R.; Vulto-van Silfhout, A.T.; Wiel, L.; Gilissen, C., et al. De Novo and Inherited Pathogenic Variants in KDM3B Cause Intellectual Disability, Short Stature, and Facial Dysmorphism. *Am. J. Hum. Genet.* **2019**, *104*, 758–766, doi:10.1016/j.ajhg.2019.02.023.
80. Guo, T.; Chung, J.H.; Wang, T.; McDonald-McGinn, D.M.; Kates, W.R.; Hawula, W.; Coleman, K.; Zackai, E.; Emanuel, B.S.; Morrow, B.E. Histone Modifier Genes Alter Conotruncal Heart Phenotypes in 22q11.2 Deletion Syndrome. *Am. J. Hum. Genet.* **2015**, *97*, 869–877, doi:10.1016/j.ajhg.2015.10.013.
81. Saez, M.A.; Fernandez-Rodriguez, J.; Moutinho, C.; Sanchez-Mut, J.V.; Gomez, A.; Vidal, E.; Petazzi, P.; Szczesna, K.; Lopez-Serra, P.; Lucariello, M., et al. Mutations in JMJD1C are involved in Rett syndrome and intellectual disability. *Genet. Med.* **2016**, *18*, 378–385, doi:10.1038/gim.2015.100.
82. Wang, L.; Yamaguchi, S.; Burstein, M.D.; Terashima, K.; Chang, K.; Ng, H.K.; Nakamura, H.; He, Z.; Doddapaneni, H.; Lewis, L., et al. Novel somatic and germline mutations in intracranial germ cell tumours. *Nature* **2014**, *511*, 241–245, doi:10.1038/nature13296.
83. Neale, B.M.; Kou, Y.; Liu, L.; Ma'ayan, A.; Samocha, K.E.; Sabo, A.; Lin, C.F.; Stevens, C.; Wang, L.S.; Makarov, V., et al. Patterns and rates of exonic de novo mutations in autism spectrum disorders. *Nature* **2012**, *485*, 242–245, doi:10.1038/nature11011.
84. Canova, C.; Hashibe, M.; Simonato, L.; Nelis, M.; Metspalu, A.; Lagiou, P.; Trichopoulos, D.; Ahrens, W.; Pigeot, I.; Merletti, F., et al. Genetic associations of 115 polymorphisms with cancers of the upper aerodigestive tract across 10 European countries: the ARCAGE project. *Cancer Res.* **2009**, *69*, 2956–2965, doi:10.1158/0008-5472.CAN-08-2604.
85. Perry, J.R.; Day, F.; Elks, C.E.; Sulem, P.; Thompson, D.J.; Ferreira, T.; He, C.; Chasman, D.I.; Esko, T.; Thorleifsson, G., et al. Parent-of-origin-specific allelic associations among 106 genomic loci for age at menarche. *Nature* **2014**, *514*, 92–97, doi:10.1038/nature13545.
86. Najmabadi, H.; Hu, H.; Garshasbi, M.; Zemojtel, T.; Abedini, S.S.; Chen, W.; Hosseini, M.; Behjati, F.; Haas, S.; Jamali, P., et al. Deep sequencing reveals 50 novel genes for recessive cognitive disorders. *Nature* **2011**, *478*, 57–63, doi:10.1038/nature10423.
87. Zaidi, S.; Choi, M.; Wakimoto, H.; Ma, L.; Jiang, J.; Overton, J.D.; Romano-Adesman, A.; Bjornson, R.D.; Breitbart, R.E.; Brown, K.K., et al. De novo mutations in histone-modifying genes in congenital heart disease. *Nature* **2013**, *498*, 220–223, doi:10.1038/nature12141.
88. Faundes, V.; Newman, W.G.; Bernardini, L.; Canham, N.; Clayton-Smith, J.; Dallapiccola, B.; Davies, S.J.; Demos, M.K.; Goldman, A.; Gill, H., et al. Histone Lysine Methylases and Demethylases in the Landscape of Human Developmental Disorders. *Am. J. Hum. Genet.* **2018**, *102*, 175–187, doi:10.1016/j.ajhg.2017.11.013.
89. Ounap, K.; Puusepp-Benazzouz, H.; Peters, M.; Vaher, U.; Rein, R.; Proos, A.; Field, M.; Reimand, T. A novel c.2T > C mutation of the KDM5C/JARID1C gene in one large family with X-linked intellectual disability. *Eur. J. Med. Genet.* **2012**, *55*, 178–184, doi:10.1016/j.ejmg.2012.01.004.
90. Abidi, F.E.; Holloway, L.; Moore, C.A.; Weaver, D.D.; Simensen, R.J.; Stevenson, R.E.; Rogers, R.C.; Schwartz, C.E. Mutations in JARID1C are associated with X-linked mental retardation, short stature and hyperreflexia. *J. Med. Genet.* **2008**, *45*, 787–793, doi:10.1136/jmg.2008.058990.
91. Tzschach, A.; Lenzner, S.; Moser, B.; Reinhardt, R.; Chelly, J.; Fryns, J.P.; Kleefstra, T.; Raynaud, M.; Turner, G.; Ropers, H.H., et al. Novel JARID1C/SMCX mutations in patients with X-linked mental retardation. *Hum. Mutat.* **2006**, *27*, 389, doi:10.1002/humu.9420.
92. Jensen, L.R.; Amende, M.; Gurok, U.; Moser, B.; Gimmel, V.; Tzschach, A.; Janecke, A.R.; Tariverdian, G.; Chelly, J.; Fryns, J.P., et al. Mutations in the JARID1C gene, which is involved in transcriptional regulation and chromatin remodeling, cause X-linked mental retardation. *Am. J. Hum. Genet.* **2005**, *76*, 227–236, doi:10.1086/427563.
93. Grozeva, D.; Carss, K.; Spasic-Boskovic, O.; Tejada, M.I.; Gecz, J.; Shaw, M.; Corbett, M.; Haan, E.; Thompson, E.; Friend, K., et al. Targeted Next-Generation Sequencing Analysis of 1,000 Individuals with Intellectual Disability. *Hum. Mutat.* **2015**, *36*, 1197–1204, doi:10.1002/humu.22901.
94. Santos, C.; Rodriguez-Revenga, L.; Madrigal, I.; Badenas, C.; Pineda, M.; Mila, M. A novel mutation in JARID1C gene associated with mental retardation. *Eur. J. Hum. Genet.* **2006**, *14*, 583–586, doi:10.1038/sj.ejhg.5201608.

95. Grafodatskaya, D.; Chung, B.H.; Butcher, D.T.; Turinsky, A.L.; Goodman, S.J.; Choufani, S.; Chen, Y.A.; Lou, Y.; Zhao, C.; Rajendram, R., et al. Multilocus loss of DNA methylation in individuals with mutations in the histone H3 lysine 4 demethylase KDM5C. *BMC Med. Genomics* **2013**, *6*, 1, doi:10.1186/1755-8794-6-1.
96. Rujirabanjerd, S.; Nelson, J.; Tarpey, P.S.; Hackett, A.; Edkins, S.; Raymond, F.L.; Schwartz, C.E.; Turner, G.; Iwase, S.; Shi, Y., et al. Identification and characterization of two novel JARID1C mutations: suggestion of an emerging genotype-phenotype correlation. *Eur. J. Hum. Genet.* **2010**, *18*, 330–335, doi:10.1038/ejhg.2009.175.
97. Vissers, L.E.; de Ligt, J.; Gilissen, C.; Janssen, I.; Steehouwer, M.; de Vries, P.; van Lier, B.; Arts, P.; Wieskamp, N.; del Rosario, M., et al. A de novo paradigm for mental retardation. *Nat. Genet.* **2010**, *42*, 1109–1112, doi:10.1038/ng.712.
98. Redin, C.; Gerard, B.; Lauer, J.; Herenger, Y.; Muller, J.; Quartier, A.; Masurel-Paulet, A.; Willems, M.; Lesca, G.; El-Chehadeh, S., et al. Efficient strategy for the molecular diagnosis of intellectual disability using targeted high-throughput sequencing. *J. Med. Genet.* **2014**, *51*, 724–736, doi:10.1136/jmedgenet-2014-102554.
99. Santos-Reboucas, C.B.; Fintelman-Rodrigues, N.; Jensen, L.R.; Kuss, A.W.; Ribeiro, M.G.; Campos, M., Jr.; Santos, J.M.; Pimentel, M.M. A novel nonsense mutation in KDM5C/JARID1C gene causing intellectual disability, short stature and speech delay. *Neurosci. Lett.* **2011**, *498*, 67–71, doi:10.1016/j.neulet.2011.04.065.
100. Adegbola, A.; Gao, H.; Sommer, S.; Browning, M. A novel mutation in JARID1C/SMCX in a patient with autism spectrum disorder (ASD). *Am. J. Med. Genet. A* **2008**, *146A*, 505–511, doi:10.1002/ajmg.a.32142.
101. Hu, H.; Haas, S.A.; Chelly, J.; Van Esch, H.; Raynaud, M.; de Brouwer, A.P.; Weinert, S.; Froyen, G.; Frints, S.G.; Laumonnier, F., et al. X-exome sequencing of 405 unresolved families identifies seven novel intellectual disability genes. *Mol. Psychiatry* **2016**, *21*, 133–148, doi:10.1038/mp.2014.193.
102. Brookes, E.; Laurent, B.; Ounap, K.; Carroll, R.; Moeschler, J.B.; Field, M.; Schwartz, C.E.; Gecz, J.; Shi, Y. Mutations in the intellectual disability gene KDM5C reduce protein stability and demethylase activity. *Hum. Mol. Genet.* **2015**, *24*, 2861–2872, doi:10.1093/hmg/ddv046.
103. Fieremans, N.; Van Esch, H.; de Ravel, T.; Van Driessche, J.; Belet, S.; Bauters, M.; Froyen, G. Microdeletion of the escape genes KDM5C and IQSEC2 in a girl with severe intellectual disability and autistic features. *Eur. J. Med. Genet.* **2015**, *58*, 324–327, doi:10.1016/j.ejmg.2015.03.003.
104. Bogershausen, N.; Gatinois, V.; Riehmer, V.; Kayserili, H.; Becker, J.; Thoenes, M.; Simsek-Kiper, P.O.; Barat-Houari, M.; Elcioglu, N.H.; Wieczorek, D., et al. Mutation Update for Kabuki Syndrome Genes KMT2D and KDM6A and Further Delineation of X-Linked Kabuki Syndrome Subtype 2. *Hum. Mutat.* **2016**, *37*, 847–864, doi:10.1002/humu.23026.
105. Guo, Z.; Liu, F.; Li, H.J. Novel KDM6A splice-site mutation in kabuki syndrome with congenital hydrocephalus: a case report. *BMC Med. Genet.* **2018**, *19*, 206, doi:10.1186/s12881-018-0724-4.
106. Banka, S.; Lederer, D.; Benoit, V.; Jenkins, E.; Howard, E.; Bunstone, S.; Kerr, B.; McKee, S.; Lloyd, I.C.; Shears, D., et al. Novel KDM6A (UTX) mutations and a clinical and molecular review of the X-linked Kabuki syndrome (KS2). *Clin. Genet.* **2015**, *87*, 252–258, doi:10.1111/cge.12363.
107. Micale, L.; Augello, B.; Maffeo, C.; Selicorni, A.; Zucchetti, F.; Fusco, C.; De Nittis, P.; Pellico, M.T.; Mandriani, B.; Fischetto, R., et al. Molecular analysis, pathogenic mechanisms, and readthrough therapy on a large cohort of Kabuki syndrome patients. *Hum. Mutat.* **2014**, *35*, 841–850, doi:10.1002/humu.22547.
108. Van Laarhoven, P.M.; Neitzel, L.R.; Quintana, A.M.; Geiger, E.A.; Zackai, E.H.; Clouthier, D.E.; Artinger, K.B.; Ming, J.E.; Shaikh, T.H. Kabuki syndrome genes KMT2D and KDM6A: functional analyses demonstrate critical roles in craniofacial, heart and brain development. *Hum. Mol. Genet.* **2015**, *24*, 4443–4453, doi:10.1093/hmg/ddv180.
109. Miyake, N.; Mizuno, S.; Okamoto, N.; Ohashi, H.; Shiina, M.; Ogata, K.; Tsurusaki, Y.; Nakashima, M.; Saitsu, H.; Niikawa, N., et al. KDM6A point mutations cause Kabuki syndrome. *Hum. Mutat.* **2013**, *34*, 108–110, doi:10.1002/humu.22229.
110. van Haaften, G.; Dalgliesh, G.L.; Davies, H.; Chen, L.; Bignell, G.; Greenman, C.; Edkins, S.; Hardy, C.; O'Meara, S.; Teague, J., et al. Somatic mutations of the histone H3K27 demethylase gene UTX in human cancer. *Nat. Genet.* **2009**, *41*, 521–523, doi:10.1038/ng.349.
111. Miyake, N.; Koshimizu, E.; Okamoto, N.; Mizuno, S.; Ogata, T.; Nagai, T.; Kosho, T.; Ohashi, H.; Kato, M.; Sasaki, G., et al. MLL2 and KDM6A mutations in patients with Kabuki syndrome. *Am. J. Med. Genet. A* **2013**, *161A*, 2234–2243, doi:10.1002/ajmg.a.36072.
112. Lederer, D.; Shears, D.; Benoit, V.; Verellen-Dumoulin, C.; Maystadt, I. A three generation X-linked family with Kabuki syndrome phenotype and a frameshift mutation in KDM6A. *Am. J. Med. Genet. A* **2014**, *164A*, 1289–1292, doi:10.1002/ajmg.a.36442.
113. Frans, G.; Meyts, I.; Devriendt, K.; Liston, A.; Vermeulen, F.; Bossuyt, X. Mild humoral immunodeficiency in a patient with X-linked Kabuki syndrome. *Am. J. Med. Genet. A* **2016**, *170*, 801–803, doi:10.1002/ajmg.a.37499.
114. Masui, D.; Fukahori, S.; Mizuochi, T.; Watanabe, Y.; Fukui, K.; Ishii, S.; Saikusa, N.; Hashizume, N.; Higashidate, N.; Sakamoto, S., et al. Cystic biliary atresia with paucity of bile ducts and gene mutation in KDM6A: a case report. *Surg. Case Rep.* **2019**, *5*, 132, doi:10.1186/s40792-019-0688-4.
115. Cheon, C.K.; Sohn, Y.B.; Ko, J.M.; Lee, Y.J.; Song, J.S.; Moon, J.W.; Yang, B.K.; Ha, I.S.; Bae, E.J.; Jin, H.S., et al. Identification of KMT2D and KDM6A mutations by exome sequencing in Korean patients with Kabuki syndrome. *J. Hum. Genet.* **2014**, *59*, 321–325, doi:10.1038/jhg.2014.25.
116. Yavarna, T.; Al-Dewik, N.; Al-Mureikhi, M.; Ali, R.; Al-Mesaifri, F.; Mahmoud, L.; Shahbeck, N.; Lakhani, S.; AlMulla, M.; Nawaz, Z., et al. High diagnostic yield of clinical exome sequencing in Middle Eastern patients with Mendelian disorders. *Hum. Genet.* **2015**, *134*, 967–980, doi:10.1007/s00439-015-1575-0.

117. Abidi, F.; Miano, M.; Murray, J.; Schwartz, C. A novel mutation in the PHF8 gene is associated with X-linked mental retardation with cleft lip/cleft palate. *Clin. Genet.* **2007**, *72*, 19–22, doi:10.1111/j.1399-0004.2007.00817.x.
118. Laumonnier, F.; Holbert, S.; Ronce, N.; Faravelli, F.; Lenzner, S.; Schwartz, C.E.; Lespinasse, J.; Van Esch, H.; Lacombe, D.; Goizet, C., et al. Mutations in PHF8 are associated with X linked mental retardation and cleft lip/cleft palate. *J. Med. Genet.* **2005**, *42*, 780–786, doi:10.1136/jmg.2004.029439.
119. Koivisto, A.M.; Ala-Mello, S.; Lemmela, S.; Komu, H.A.; Rautio, J.; Jarvela, I. Screening of mutations in the PHF8 gene and identification of a novel mutation in a Finnish family with XLMR and cleft lip/cleft palate. *Clin. Genet.* **2007**, *72*, 145–149, doi:10.1111/j.1399-0004.2007.00836.x.
120. Nava, C.; Lamari, F.; Heron, D.; Mignot, C.; Rastetter, A.; Keren, B.; Cohen, D.; Faudet, A.; Bouteiller, D.; Gilleron, M., et al. Analysis of the chromosome X exome in patients with autism spectrum disorders identified novel candidate genes, including TMLHE. *Transl. Psychiatry* **2012**, *2*, e179, doi:10.1038/tp.2012.102.
121. De Wolf, V.; Crepel, A.; Schuit, F.; van Lommel, L.; Ceulemans, B.; Steyaert, J.; Seuntjens, E.; Peeters, H.; Devriendt, K. A complex Xp11.22 deletion in a patient with syndromic autism: exploration of FAM120C as a positional candidate gene for autism. *Am. J. Med. Genet. A* **2014**, *164A*, 3035–3041, doi:10.1002/ajmg.a.36752.
